# Are we bad winners? Public understandings of the United Nations’ World Happiness Report among Finnish digital media and their readers

**DOI:** 10.1177/09636625221132380

**Published:** 2022-12-05

**Authors:** Jennifer De Paola, Anna-Maija Pirttilä-Backman

**Affiliations:** University of Helsinki, Finland

**Keywords:** lay expertise, media representations, metaphors, public understanding of science, social representations

## Abstract

In this research, we investigate the public understanding of the World Happiness Report within the context of its highest-ranking country: Finland. We analyse how two actors, Finnish online media and their readers, understood the publication as well as the concept being measured: happiness. Digital media adopted an ambivalent stance towards both the World Happiness Report (‘sports victory’ vs ‘societal problems’) and the concept of happiness (‘reticence to define happiness’ vs ‘secrets of Finnish happiness’). Readers agreeing with the World Happiness Report define Finland as an ‘almost utopia’ while readers disagreeing with the World Happiness Report, in addition to presenting a reversed image of Finland (‘almost dystopia’), further justify their distrust towards the World Happiness Report by attacking the publication, its authors and the participants (Finns). Both actors carefully construct their understanding of happiness to fit their arguments aimed at the glorification/scandalization of the World Happiness Report.

## 1. Introduction

‘Finland is the happiest country in the world, and Finns aren’t happy about it’, announces *American Scientific* in 2018, shortly after Finland was ranked first in the United Nations’ World Happiness Report (WHR). As Finland upholds its top spot in the ranking for the fifth year in a row (Helliwell et al., 2022), other news headlines published in Finland and around the world reiterate the same narration: the Finnish public is simply puzzled at Finland gaining the title of *happiest country in the world*. But why would (some) Finns discountenance their country’s positive performance in the ranking?

In the present article, we offer insight into the everyday understandings of the WHR in terms of an international ranking assessing a highly abstract yet ubiquitous concept: happiness. We start by presenting research on international rankings, debates triggered by those rankings in the public, the nature of those debates, and contestation of science and expert knowledge in contemporary societies. We then present Social Representation Theory (SRT), the framework adopted in the presented study due to lay understandings of scientific or novel issues being one of its key features, and the research questions posed in our study. Next, we introduce the methods and material employed in the study. Finally we present our findings, their interpretations and implications.

### Encounters between science and common sense

When making sense of complex or novel scientific issues, such as genomics or nanotechnology, both media and the general public rely on expert knowledge because they (often) lack the domain-specific knowledge needed to assess claims pertaining to these fields of expertise (Ahola, 2016). Unlike genomics or nanotechnology, happiness is a concept for which the average citizen has developed some sort of understanding without having to rely on expert knowledge, simply because of the importance people attach to happiness and its achievement (Diener, 2000). The puzzled reactions of Finns towards Finland’s performance in the WHR suggest that the definition of happiness endorsed in the report and the results may not be (fully) accommodated by the general public.

A lack of consensus on how to consistently define and measure happiness also exists among well-being scholars (Carlquist et al., 2017), although we can identify two main strands of research. First, many studies identify happiness with Subjective Well-being (SWB; e.g. Diener, 2009), a model encompassing cognitive assessment of life satisfaction and frequencies of positive and negative affect. Second, others (e.g. Waterman, 2008) have proposed the concept of eudemonia, which understands happiness in terms of the meaning of life. In addition to these two main fields of happiness research, Delle Fave et al. (2016) note that it is not uncommon for studies solely based on data collected through the Cantril Ladder to use the term ‘happiness’. The instrument features a single item asking respondents to rate how close their current life approximates the ‘best possible life’ on a 0–10 scale (Kilpatrick and Cantril, 1960).

This is the case with the WHR, the annual publication produced by the United Nations Sustainable Development Solutions Network (SDSN) since 2012 (Helliwell et al., 2022). The report uses data from the Gallup World Poll to rank selected nations (~150) according to their response to the Cantril Ladder. In addition, six factors – gross domestic product (GDP), life expectancy, generosity, social support, freedom and corruption – are used to explain differences in the ranking of the countries. The data also include measures relating to how often participants experience positive and negative emotions, which, however, do not affect the ranking.

International rankings are more popular than ever among the general public (Mau, 2019). A wealth of literature exists regarding their social impact and surrounding public discourse (e.g. Espeland and Sauder, 2007, 2016; Esposito and Stark, 2019; Mau, 2019, 2020).

Ratings and rankings are widely prominent in contemporary societies due to their ability to provide concise information on a particular area of interest in a fast yet easy-to-comprehend manner, even for someone who is not an expert in the specific field considered (Martens and Niemann, 2013). Due to their evaluative nature, which attributes specific positions in a symbolic hierarchy, rankings spur public and political debate on potential policies and foster a climate of competition.

The reception of an international ranking by the public is not passive but instead involves accepting, rejecting or re-negotiating a message (Pons, 2012). Steiner-Khamsi (2003) noted that rankings can result in three extreme types of public debate and policies: *scandalization, glorification* and *indifference*. Scandalization consists of highlighting the weaknesses of one’s own national systems and usually occurs when the country scored lower than expected. In contrast, glorification is mostly elicited in the case of excellent performances, and enacted through celebration and acclamation of one’s country in relation to the object of the ranking. Finally, indifference occurs in instances in which the object of the international ranking is not particularly salient for a specific country. In spite of what is expected according to Steiner-Khamsi’s model, there are instances in which extremely low or high performances are not followed by, respectively, scandalization and glorification.

For example, in spite of Finland’s achievements in the Programme for International Students Assessment (PISA), in the Finnish media ‘a considerable number of arguments were evinced, attempting to show that there are still problems in Finnish education’ (Rautalin, 2018: 1780), eliciting a public debate closer to the tones of scandalization, which is usually engendered by scores that are lower than expected.^1^ As demonstrated earlier (Rautalin and Alasuutari, 2007), this was achieved through creative rhetorical strategies specifically aimed at *attacking* the validity and reliability of the statistical results. Strategies included pointing out shortcomings related to study design and method and highlighting societal problems, such as a high incidence of mental health-related issues among young people and burnout among teachers. Bringing up statistical evidence related to mental health of specifically young people and teachers vis-a-vis the positive performance in the PISA study is a way to draw attention to a seemingly irreconcilable *contradiction* stemming from different assessments targeting the same segment of society. Rautalin (2018) interpreted the rhetorical strategies as an attempt to claim that Finnish education is not problem-free, and that in spite of PISA, a change in the national education policies is much needed. A similar kind of trend has been documented in Japan, another high-ranking country (Takayama, 2008).

Instances of scientific claims being challenged have often been studied in the context of disagreements between experts. There are several models that explain how laypeople justify their challenges towards expert knowledge. In their study on laypeople’s thoughts and opinions on expert disagreements regarding food additives, Kajanne and Pirttilä-Backman (1999) reported three main explanations for the disagreement. While the first category, *general difficulty in obtaining scientific knowledge*, related to the research itself, the second and third categories, *various interest-related reasons* and *calibre and personal background of experts*, directly referred to the experts. Similarly, Barnes et al. (2018) distinguish between *attacks on the empirical basis of the claim* and *ad hominem attacks*, namely on the scientists who made the claim. Gierth and Bromme (2020) focused on science-critical online user comments attacking controversial scientific claims (homoeopathy, genetically modified organisms, refugee crime and childhood vaccinations). The authors revealed that online comments challenged scientific claims based on four elements: *thematic complexity, the employed research methods, expertise* or *the motivations of the researchers*. Thus, research on challenges towards scientific claims shows that the general public are rather versatile in their argumentations. Their modes of challenge include statements directed at both empirical work and experts.

### Social Representation Theory

While the public understanding of science is of interest for a number of disciplines, there is no unifying paradigm explicating the relation between science and society at an interdisciplinary level (Kronberger, 2015). However, two major paradigms are often proposed to illustrate possible approaches (Ahola, 2016). The deficit model entails one-way communication between the scientist possessing the knowledge and the general public receiving the information: experts provide content to fill the ‘knowledge vacuum’ of the public (Miller, 2001: 116). In contrast, the contextual/lay expertise approaches acknowledge that ‘individuals do not simply respond as empty containers to information, but rather process information according to social and psychological schemas that have been shaped by their previous experiences, cultural context, and personal circumstances’ (Brossard and Lewenstein, 2010: 13).

In the present study, we seek to analyse public understanding of the WHR among the general public in Finland from two perspectives: digital media and their readers. We rely theoretically and methodologically on SRT (Moscovici, 2008 [1961]), a framework that aligns closely with the contextual/lay expertise approaches to the public understanding of science. Social representations can be described as ‘the ways individual think, interact with others and shape social objects in their interaction with the local world’ (Wagner, 2020: 1). In the most commonly known definition, Moscovici (1973: xiii) describes social representations as ‘A system of values, ideas and practices’, fulfilling two functions: orienting us in the social world and enabling communication and social interaction between individuals and groups. SRT acknowledges that scientific theories may circulate among the lay public (Wagner, 2007); it is thus an optimal approach for surmising what happens when an international ranking measuring ‘happiness’ encounters common sense. Initially developed by Serge Moscovici in order to investigate the reception of psychoanalysis in French society during the 1950s (Moscovici, 1984, 2008 [1961]), SRT enables to surmise how common-sense thinking unfolds in the face of new abstract knowledge, which is transformed into something familiar and comprehensible through the processes of anchoring and objectification. Anchoring involves classifying and naming new concepts according to previous/existing knowledge. For example, in Moscovici’s (2008 [1961]) classic study on social representations of psychoanalysis, he found that Catholics in France anchored psychoanalysis to the concept of confession. Objectification, working in synergy with anchoring, tempers the impalpable nature of unfamiliarity by assigning a concrete image to an abstract concept; the result of objectifying can be an icon, metaphor or trope. For example, in a study on genetically modified organisms (GMOs) (Wagner and Kronberger, 2001), participants employed images of scientists injecting tomatoes to lend more concrete form to the complex notion of GMOs.

When the information around which social representations are created entails a scientific object, it is reasonable to extend the investigation to different arenas of communication. In Farr’s (1993) words, social representations of scientific objects ‘are to be found in the media as well as in people’s minds, and need to be sampled in both locations’ (p. 189). This approach has been adopted, for example, by Joffe and Haarhoff (2002) in their work on social representations of Ebola in the United Kingdom. They analysed British broadsheets and tabloids and interviews with their readers to examine how the general public made sense of this illness. Their results showed that while there were certainly similarities between media and readers’ discourses and understandings about Ebola, an interesting difference existed. While newspapers strived to make Ebola ‘concrete’ to justify writing multiple articles about it, readers constructed Ebola as a fiction-like illness, thus detaching from the possible threat posed by the disease.

In recent years, commenting spaces provided by online news websites offer readers a direct way to participate in discussions. Differently from traditional mass media and expert arenas, which are characterized by high barriers to communication (Schmidt et al., 2013), the current mass media-induced arena is characterized by low barriers to communication, permitting laypeople to express their views following initial journalistic input (Lörcher and Taddicken, 2017). In spite of readers’ involvement in shaping media content, there is surprisingly little research on the ‘patterns of audience comments to online newspaper stories or other online content’ (Peters and Dunwoody, 2016: 899) and even fewer studies (e.g. Lörcher and Taddicken, 2017) that consider both online news articles and their readers’ comments. By examining how Finnish digital media and their readers have received and made sense of Finland’s ranking in the WHR, the present study aims to participate in the wider theoretical debate contemplating different modes in which scientific claims can be challenged (Gierth and Bromme, 2020). Following closely the user comments engendered within the corresponding media narrative, we strive to map how an international report measuring a concept as abstract as happiness is unpacked in the digital media as well as in digital media-induced arenas. We pose two research questions:

(1) How are the United Nations’ WHR in general and Finland’s performance in the ranking in particular received and made sense of by Finnish digital media and their readers?
(2) When the media and their readers make sense of WHR, what kind of understandings of the notion of happiness do they use?

## 2. Methods and material

Our main aim was to analyse Finland’s national news and general public discussions, anchorings and objectifications that followed the yearly publication of the WHR results. As our study is focused at the national level, we selected widespread digital media platforms that allow readers to post comments online related to specific news. Three of our sources are newspapers also offering printed versions. We selected two ‘broadsheet-quality’ sources, the national newspaper *Helsingin Sanomat* (HS) and the online news page *Yle uutiset* (YLE), and two tabloid newspapers, *Ilta-Sanomat* (IS) and *Iltalehti* (IL).^2^

Articles were found with the keywords ‘World Happiness Report’ and ‘onnellisuusraport*’, the equivalent of WHR in Finnish language. As Finnish is an agglutinative language, we searched for the term in the abbreviated form in order to capture possible inflections. We searched for these terms in articles published by the four news sources between March 2018 and March 2020. Comments were selected taking into account the saturation point of the data, which set our cut-off point at N = 250 comments. In light of ethical concerns related to analysing data generated within online communities (Sipes et al., 2020), we took into account the privacy of online users. All comments were publicly available, and thus considered suitable for research purposes. In addition, although comments are always published under nicknames rather than under the readers’ official names, we removed them along with any other reference to users’ personal life.

Overall, we collected 25 newspaper articles and 2290 comments from online readers. Specific numbers and respective word counts of the broadsheets and tabloid articles/comments are reported in Table 1.

**Table 1.** Number and word count of broadsheet and tabloid articles and related comments.

	News_N	News_word count	Comments_N	Comments_word count
Broadsheet_HS	8	3638	341	18,876
Broadsheet_YLE	7	3315	441	21,772
Broadsheet_tot	15	6953	782	40,648
Tabloid_IS	5	1694	892	33,831
Tabloid_IL	5	934	506	9559
Tabloid_tot	10	2628	1398	43,390

HS: *Helsingin Sanomat*; YLE: *Yle uutiset*; IS: *Ilta-Sanomat*; IL: *Iltalehti*.

Our analysis was guided by SRT conceptual tools (anchoring and objectification) in combination with the basic tenets of Grounded Theory (Charmaz, 2006; Strauss and Corbin, 1990), which entails building the analysis ‘from the ground up’ (Charmaz, 2006: 51) based on the data at hand, keeping an open mind towards different directions. Analysis started with ‘open coding’, developing simple and concise codes and possible categories for grouping them *inductively.* While inductive logic allows approaching the data with previous findings from the scholar’s field, greater emphasis is placed on remaining open to new concepts drawing from the data itself. We often adopted journalists’ and readers’ expressions as they were (*in vivo* coding; e.g. Charmaz, 2006). Following the identification of recurring and significant codes, we focused on codes related to the WHR and the concept of happiness, refining the emerging categories (and possible sub-categories), and looking at how they were interconnected. We complemented this phase with the theoretical integration of the tools provided by SRT, which meant extracting anchorings and objectifications of the WHR (RQ1) and the concept of happiness (RQ2) from the data.

Following Moscovici’s (1984) presentation of the processes involved in the formation of a social representation, we considered anchoring in terms of a process involving naming and classifying novel or complex phenomena within the backdrop of existing understandings and classifications, and objectifications as all the possible images, icons, symbols, metaphors, tropes or people (Sakki, 2010) used to lend concrete forms to the otherwise abstract notions of happiness and its measurement. When looking for objectifications, we included in the material actual pictures presented in the digital media articles, as well as images evoked verbally by the journalists or readers.

Objectifications were identified directly from text (or images). Anchorings could be extracted directly from text (as shown in the examples below), but they can also appear implicitly in the language. In those cases, we utilized the concept of anchoring in an interpretative way (for a similar approach, see Pirttilä-Backman et al., 2017), taking into account the sociocultural frame of reference and our own cultural knowledge. Supplemental Material presents a more in-depth example of the analytical process.

## 3. Results

In the ‘Results’ section, we first present how the WHR is received and made sense of from the perspectives of the two actors: digital media and their readers. Second, we present how the two actors make sense of the WHR in light of their own understandings of how happiness should be defined and measured.

In this section, we focus on anchorings and objectifications of WHR and happiness, and direct the reader to the ‘Discussion’ section for a more extensive interpretation of their functions. We conclude the ‘Results’ section with an illustration summarizing our main findings (Tables 2 and 3).

**Table 2.** Representation of the WHR: objectifications, anchorings, and their functions.

Actor	Representation of the WHR	Anchorings	Objectifications	Functions
Media	Ambivalent	Sports news vs societal problems	Winner (e.g. ‘Finland won again. Finland is the happiest country in the world’ (Yle, 2019)) vs contradicting national welfare statistics (e.g. ‘How can Finns be both depressed and happy? (Yle, 2018))	Create momentum; appeal to a wider audience
Readers Public	Positive	Almost utopia	Clean water, winning lottery, utopic locations [e.g. ‘[. . .] If only we could enjoy all the good things we have! In spite of sometimes being controlling and having strict rules, Finland is still a Lintukoto* compared to many other countries’ (Yle, 2019)] *In Finnish mythology, Lintukoto is a mythical place located, where the sky and the surface of the earth meet	Reinforce national identity and prove efficiency of Finnish system or society
	Negative	Almost dystopia	Immediate threat in the form of a dangerous beast about to attack, building about to collapse, etc. (e.g. ‘Finnish society is like a mouldy house: maybe someone is still celebrating in the attic, but the lowest floors have been left in such a state that no one can remain healthy there anymore’ (Yle, 2019))	Appeal to sense of threat and fear for the status quo and future; impute negative connotations to Finns and FinnishnessAppeal to mistrust towards the measure (attacks on research method/process)
			Bread queue	
			Dictatorship	Appeal to mistrust towards the government/researchers and their relationship (attacks on motivations of the researcher)

WHR: World Happiness Report; YLE:*Yle uutiset*.

**Table 3.** Representations of happiness: objectifications, anchorings and their functions.

Actor	Representation of happiness	Anchorings	Objectifications	Function
Media	Ambivalent	Complexity vs Finnish happiness	‘Secrets’ to happiness (e.g. ‘The one who has happiness should hide happiness. By following this old advice, we give our fellow humans less cause for envy, which can therefore increase everyone’s happiness’ (HS, 2018))	Different representations of happiness serve the function of justifying the ambivalent, positive or negative stance towards the WHR
Readers Public	Based on internal factors	Agency	Blacksmith, baker (e.g. ‘Personally, I think we have a lot of good things that lead to happiness. You just have to be your own happiness blacksmith’ (YLE_2019))	
	Complex and based on emotions	Positive emotions	Missing smile; happy ‘other’ (e.g. ‘A Finnish child gets angry and doesn’t feel unless he get the latest model iPhone as a Christmas present. A boy living in a Brazilian slum is extremely happy if he gets to kick a donated soccer ball’ (Yle, 2019))	

HS: *Helsingin Sanomat*; WHR: World Happiness Report; YLE: *Yle uutiset.*

### Representations of the WHR

#### Digital media perspective: ‘Sports victory’ versus ‘societal problems’

There is great variation in the accuracy level of how our news sources present data and methodology behind the WHR. While some articles present the ranking as resulting from the Cantril Ladder, in other instances the focus is on the six factors (levels of GDP, life expectancy, generosity, social support, freedom and corruption), while the Cantril Ladder – the measure the report is based on – is ignored.

**Figure 1.**
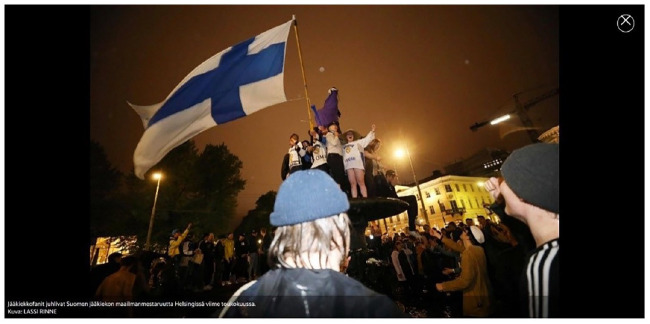
Image of ice-hockey victory celebration accompanying an article about the WHR. Reprinted with kind permission of Lassi Rinne/*Ilta-Sanomat*.

A common feature in many of the stories is the ample space given to the ranking itself and, more specifically, to Finland’s top position. The way journalists describe the WHR is highly reminiscent of world contests, and anchored to sports news: ‘Finland won again. Finland is the happiest country in the world’ (YLE, 2019).^3^ In one instance, University of Oxford Professor De Neve likens the countries’ rankings in the pole positions to those of Premier League football teams. The top-ranking position in the WHR is thus objectified as a ‘title’ to win, to be ‘crowned with’ or ‘chosen for’. The news is often accompanied by images of Finnish flags and people smiling and/or celebrating, mirroring the victory-like language in the texts, as shown in Figure 1.

Together with the celebratory tone of the news, most articles create momentum around the surprise effect elicited by the WHR results:

Finland has been chosen as the world’s happiest country – ‘Sounds weird!’ (IL, 2018)

This is followed by an attempt to contextualize these surprising results, trying to make sense of possible arguments in favour and disfavour of Finland’s position as a winner. In support of the WHR ranking, some articles resort to emphasizing qualities intrinsic to Nordic welfare societies, such as the free education system and healthcare, equality, and trust. However, depictions of Finland in terms of well-functioning welfare are carefully balanced with negative illustrations, anchored to darker aspects of Finnish society and objectified with statistics on Finland’s grim achievement in a variety of societal issues: ‘There is also a lot of research data on issues [related to] Finnish society: depression, loneliness, inequality, insecurity and poverty. (IS, 2019)

#### Readers’ perspective: ‘Utopia’ versus ‘dystopia’

Readers’ comments on the position of Finland as the winner appear anchored to Finland as ‘almost utopia’, on one hand, and ‘almost dystopia’, on the other.^4^

In the first instance, readers show pride towards their country’s performance in the WHR, saluting the news with the same cheering attitude and congratulatory tone customary in the afterglow of a world-contest victory: ‘Well done, Finland and Finns! (HS, 2020); Congratulations to the Maiden of Finland’ (IS, 2019).^5^

Utopian depictions are objectified through the widely shared metaphor of having won the lottery by being born in Finland (*lottovoitto*) as well as through the use of several utopia proxies, such as paradise (*paratiisi*), Finnish mythological locations like ‘island of bliss’ (*lintukoto*) and the Finnish synonymous ‘earthly paradise’ (*onnela*). In spite of the utopia-like connotations attributed to Finland, the readers often soften their construction by reinstating that, even for Finland, there is some room for improvement and no country is absolute perfection. However, Finland remains the best country in the world – or the universe – where one can live.

The utopic quality of Finland is supported through concrete examples of qualities inherent to Finnish society: welfare, high trust, safety and low corruption, and through the more tangible depictions of Finnish nature as unspoiled, untouched and clean. This last characteristic is often objectified with the recurring image of drinkable water, which makes Finland different from other countries: ‘We have clean water, even for flushing the toilet. There is no need to leave with a pail on your head in the morning to fetch water from a well many kilometres away’ (IS, 2019).

The largest proportion of comments, however, paints a picture of Finland with strong negative connotations. The dystopia-like scenario is positioned in the present and near future, in stark contrast with a past where safety, trust and welfare were still qualities to be found in Finnish society:

The direction in Finland is the same, but honestly back in the ’70s it was different here in the countryside, there was no need to lock the doors, you could just place a brush or broom leaning on the door if you were not at home and this is true because I remember it well myself. Now things are not so good anymore. (IS, 2019)

References to the future are used to construct the image of a country on the verge of catastrophe and under immediate threat, which is often objectified as a ‘ship about to sink’, a ‘building about to collapse’, a ‘riot about to explode’ or a ‘dangerous being’. These images objectify profound dissatisfaction with the status quo vis-a-vis an idealized past, where ‘children could be left to sleep outside’ and ‘front doors were left unlocked’ (IS, 2019).

Arguments given in support of this dystopian scenario refer primarily to political and financial aspects, and to specific issues notoriously plaguing Finnish society: high rates of depression, a high consumption of antidepressants and alleged high rates of suicide. Discourse around financial challenges is generally accompanied by concerns regarding the research validity and the extent to which the sample the report is based on can be considered representative of the general population. This aspect is objectified with the image of citizens standing in ‘bread queues’. The argument is that researchers have failed to include in their sample low-income recipients, who often find themselves relying on charitable organizations to receive free food, as they struggle to make ends meet on a meagre salary or income support:

Depends on whom you ask. If you ask influential people and politicians, surely they are happy as they live in abundance and make the weaker ones miserable as they get happier. If the low-income people are asked, then hunger and lack of money, etc. do not make a person happy, on the contrary, happiness is far from those who cannot afford anything and have to go to the bread queue so that their children can get bread at least once a day. (IS, 2018)

Concerns related to research validity are also made sense of in terms of the WHR authors’ inability to understand cultural aspects when dealing with Finnish participants and in terms of intentional fabrication of biased data. In the first instance, readers show perplexity towards the WHR authors’ inability to understand the Finnish context and capture valid responses, bypassing social desirability-related issues: ‘Every Finn has already learned how to answer these questions. So the answer is not how you feel but how the questions should be answered. And it has nothing to do with the truth’ (HS, 2019). ‘How can Finland be the happiest country on earth? Statisticians should move here and experience this for a few years’ (IS, 2019). In the latter case, readers question the validity of the report suspecting the WHR results have been forged by the Finnish government to present a positive image of the country and thus gain the approval and support of Finnish citizens. Seen here is profound distrust towards the Finnish government and belief of scientific information being manipulated to attain political goals:

Obviously, some research invented and carried out by the Finnish state, a bit like the GDR^6^ in the past was chosen like the best place to live in the world for many years in a row, and the research was carried out by Stasi. (IL, 2020)

In these instances, the validity of the WHR is repeatedly derogated by referring to it as a ‘hoax’ and ‘propaganda’, and objectified through metaphors likening Finnish society to dictatorships or a non-democratic government – ‘Communist Cuba’, ‘North Korea’, ‘the GDR’ and so on.

### Representations of happiness

#### Digital media perspective: ‘Reticence to define happiness’ versus ‘secrets of Finnish happiness’

The majority of the news articles (especially the lengthier ones) introduce their understanding of happiness to give context to and make sense of the WHR. In all four media sources, the consensus is that happiness is a fuzzy concept anchored to complexity, resistant to any comprehensive definition and therefore hard to measure:

Happiness is an abstract concept that is not easy to study in a reliable way –not all of its elements are understood precisely. Even though the numerical indicators of happiness are high, material well-being alone is not enough for getting a sense of happiness. Happiness is subjective, each person’s own emotional experience lies between the ears of the respondent. The feeling of happiness varies. (IS, 2019)

The notions of happiness the journalist presents in this extract are reproduced, albeit with different wordings and variations, in several other articles across the four news sources considered.

A second way in which happiness is discussed focuses instead on finding answers to the question of ‘what makes Finns the happiest people in the world?’, anchoring the general notion of happiness to the more specific notion of ‘Finnish happiness’, objectified through the image of ‘secrets behind Finnish happiness’:

The ability of Finns to express genuine emotions indicates high levels of trust. A society that trusts the authorities and other people is happy. If we befriend someone, they become a real friend to us. It is important for Finns not to leave a friend. That is one of the secrets of happiness. (YLE, 2019)

#### Readers’ perspective: ‘Happiness as emotions’ versus ‘happiness as agency’

Readers endeavour to discuss the WHR results in light of their own understanding of what the concept of happiness entails and – to some extent – how it should be measured. Overall, readers from all four newspapers share the understanding of happiness as a complex, multi-layered concept which can nevertheless be evaluated by observing the (lack of) expression of positive emotions among others: ‘If Finns are the happiest people in the world, why doesn’t anyone smile here? Explain that, if you can’ (IS, 2019). In addition to agreeing on Finns performing poorly in the display of positive emotions, readers concur in constructing a more concrete image of *others* who, unlike Finns, manage to rise above their unprivileged position and experience as well as display positive emotions. The happy ‘other’ is represented through a variety of nationalities, usually from countries and continents that are less wealthy and less modernized, compared with Finland, such as Africa or Bangladesh:

Now compare, for instance, those Bengalis who knit our T-shirts. While the people from that culture are smiling, for us the corners of the lips just won’t lift up. (IS, 2019)

Finns are not happy, I have met the happiest people in Africa, they have different values compared to us. (YLE, 2019)

In contrast, Finnish people are described as people who ‘are jealous, laugh, mock and snap at each other, derogating them’. (HS, 2018)

Happiness is also often represented in terms of something to be actively pursued: ‘I personally think we have a lot of good things leading to happiness. One just need to be their own happiness/fortune blacksmith’ (YLE, 2019). In this sense, it seems that whether Finland is (or is not) the happiest country in the world is deemed of little or no importance, as the individual alone is responsible for being their own happiness ‘blacksmith’ or ‘baker’.

## 4. Discussion

Our study aimed at investigating the public understanding of the WHR, within the context of the highest ranking country in the report: Finland. We approached this endeavour on two levels. While our first research question looked at how the Finnish public received, discussed and understood the report, the second research question asked how the Finnish public understood and defined the object of the specific ranking: happiness.

Our results showed that there is a difference in the way our two actors – digital media and their readers – understand the WHR and the concept of happiness.

### Making sense of the WHR

#### Digital media perspective

Digital media present an ambivalent stance towards the ranking, carefully balancing between the use of celebratory prose and concerns regarding societal problems, which challenge the results obtained in the report.

Anchoring the WHR to news reporting in the realm of sports, digital media introduce the WHR in terms of a competition in which Finland keeps performing exceptionally well. This parallel with the sports world is further objectified through the use of competitive terms (‘Finland won’ or ‘champion countries’), through images depicting Finnish ice hockey players or their fans, and through means of explicit competitive comparison. References to the realm of sports in the context of international rankings have been noted before. For example, Landahl (2018) reported how several Swedish newspapers described an international large-scale assessment concerning educational achievement employing competitive rhetoric, evident in the written text (e.g. by referring to the ranking as ‘knowledge Olympics’), and in accompanying illustrations. In one news story, for instance, pupils were depicted as athletes running on tracks labelled with different school subjects.

We believe that the sports/competition rhetoric serves to reinforce and give context to Finland’s pole position. By placing emphasis on the competitiveness characterizing international rankings, journalists are able to anchor the WHR to the more accessible notion of winning in sports. Due to its cultural relevance in Finland, we interpreted the direct reference to ice hockey as extending beyond the more obvious invitation to bask in reflected glory (Cialdini et al., 1976), encompassing elements of appeals to national identity (Hakoköngäs, 2017).

Similarly to Finland’s performance in the PISA study (Rautalin and Alasuutari, 2007), along with competitive/sports prose and the acknowledgement of positive aspects of Finnish society, digital media concur in highlighting how the WHR results are in stark contrast with other statistics concerning well-known societal problems (such as poverty, mental health and substance abuse issues). We propose that there may be a twofold purpose in mentioning societal problems. First, by strategically comparing rankings in desirable statistics with undesirable ones (e.g. violence or mental health statistics), journalists are able to craft a discourse centred on paradox, where tension between high levels of well-being and ill-being confer momentum to the stories and their headlines. As noted by Guenther and Ruhrmann (2016), calling attention to uncertainty and contradictory findings is a factor that journalists consider newsworthy. Second, by acknowledging the merits of the WHR while at the same time leaving space for discussing ‘contradicting statistics’, the same writer is able to craft a story appealing to a wider audience, so that different readers (dis)approving of the report can relate to the discourse.

#### Readers’ perspective

The discourse around WHR and the concept of happiness appears more polarized among readers. Readers who relate positively with Finland’s performance in the WHR anchor the notion of the ‘happiest country in the world’ to the notion of an almost utopia, mapping a picture of Finland as a place bordering on perfection. This is done by compiling lists of merits/positive aspects related to Finland, often complemented with conclusions such as ‘and the lists could go on forever’. This discourse, far from being a mere dry compilation of societal aspects, is suffused with metaphors and vibrant images. Specifically, three objectifications stand out from our analysis. First, the idea of utopia is rendered more vivid through metaphors comparing Finland with culturally known mythological places (e.g. an island of bliss). Second, Finnish citizenship, granting access to life in a country where welfare and the education systems are nearly perfect, is objectified with the image of winning the lottery. A recurring metaphor implying pride and gratitude for being a Finn, the image can be found in everyday speech as well as in popular culture. For example, *Lottovoitto on syntyä Suomeen* [‘It is a win in the lottery to be born in Finland’] is a song by one of the most popular singers in Finland, Kari Tapio, dating back to 1992.

Finally, abstract connotations of purity and cleanliness attributed to Finland are objectified with the recurring image of clean and drinkable water, which distinguishes Finland (and Finnish nature) from other countries. Far from being a casual or neutral choice of image, water has been shown to be connected with notions of cleanliness, purity and health in the minds of the general public (Farr, 1993). Together, mythological places of bliss, winning the lottery, and clean water appeal to national identity and construct a more observable portrayal of Finnish society.

Readers who relate negatively with the WHR results mirror in reverse the reception of the report shown above, warping the title of happiest country in the world into a humoristic yet dramatic notion of Finland as dystopian. We have identified three ways in which readers objectify this reversed representation.

First, in stark contrast with the mythological places presented above, readers objectify the idea of impending threat with a series of catastrophe-related images. These fantasy-like scenarios, appealing to fear for the impending threat and longing for a better time, aim at justifying dissatisfaction with current political, financial and societal aspects of the country and the need for change.

Second, the image of low-income residents standing in a ‘bread queue’, mentioned throughout all threads examined, lends a concrete form to the (alleged) failure to construct a valid sample representative of the general Finnish population. This image serves to appeal to a general sense of distrust towards the research design. Besides concerns related to the generalizability of the sample, readers also raise questions about the ability of the researchers to overcome social desirability effects and concerns towards possible differences in emic understandings of both happiness and the scale numbers.

The third argument in support of the WHR being a fallacy is the alleged fraudulent involvement of the Finnish government with the United Nations in order to obtain a favourable outcome in the ranking, in order to present a positive image of Finland. By likening the Finnish government to dictatorships (Communist Cuba, North Korea, the German Democratic Republic, etc.) readers are able to lend a form to their allegations, proving how instances of government manipulation have taken place in a not-so-distant past as well as in the present.

### Making sense of happiness

Our second aim was to investigate the Finnish public’s understanding of the concept of happiness, as this is the concept the United Nations chose to label the object of their assessment.

While there are differences in the way the concept of happiness is represented by media (difficult to quantify vs accessible through ‘secrets’) and by the readers (emotions vs agency), we believe these representations serve similar functions: employing a concept of happiness fitting specific stances towards the WHR. From the digital media perspective, the concept of happiness presented enables Finnish media to once again present multiple perspectives appealing to a wide audience and participate in the discourse around the WHR. As regards the readers, both positive and negative stances carefully construct their understanding of happiness to fit within their arguments aimed at the glorification (positive stance) and scandalization (negative stance) of the ranking.

#### Media perspective

From the digital media perspective, the concept of happiness is frequently discussed in the attempt to better understand the results of the WHR. The concept is approached from two different angles: first, the narrative put forward in the articles constructs happiness in terms of complexity and abstraction and as encompassing positive emotions, which according to most journalists are not taken into consideration in the WHR. From this perspective, the concept of happiness adopted in the WHR is overly simplified and lacks an important component of happiness (emotions). Side by side with this approach, digital media endeavour to illustrate the possible secrets behind Finnish happiness, thus overcoming the abovementioned challenges in (a) defining happiness and (b) trusting a measure of happiness which does not include emotions. Similarly to the public discourse on the excellence of the Finnish education system and its ‘secrets’ ensuring the top ranking of Finland in the PISA study (Rautalin, 2018), digital media attempt to offer an explanation for Finland’s performance in the WHR while also instilling doubts regarding what exactly is being measured in the report. Thus, we suggest that the digital media’s understanding and sense-making of happiness are characterized by ambivalence. We deem it possible that once again, the ambivalent stance adopted towards the concept of happiness may serve a twofold function: first, casting doubts on whether the WHR does in fact measure happiness – which is complex and encompasses emotions. Second, constructing a possible explanation of ‘what makes Finns happy’ enables digital media to participate in the revelation of the secret behind so-called Finnish happiness.

#### Readers’ perspective

The readers’ views on happiness appear to be split into two groups: one constructing happiness in terms of positive affect and the other one constructing happiness in terms of agency. Readers making sense of happiness in terms of positive affect are specifically concerned with a physiological display of positive emotions, a way of making sense of happiness which previous studies have shown resonates with the question of ‘what does happiness look like?’ (De Paola et al., 2021). For these readers, happiness is seen as a trait that concurs with constructing and defining national identity: while a lack of emotional expression (e.g. various references to Finns not smiling often) defines Finns, other nationalities are constructed as ‘happy others’, who are capable of experiencing and displaying positive emotions. Interestingly, the ‘happy other’ is objectified with images of individuals from countries with a lower income carrying out mundane activities. The message is clear: living in a more developed country and/or enjoying high income does not guarantee that citizens will experience positive emotions.

From the perspective of readers making sense of happiness in terms of agency, happiness is mainly an individual matter. References to ‘being your own your happiness blacksmith’, for example, serve to present a concrete image: scoring high as a nation in a happiness ranking, although commendable, only goes as far as offering the potential to be happy, while the rest depends on people themselves. The idea of happiness as something that can be actively pursued through the right choices and actions appears to be very common among Western societies and in Finnish society in particular (De Paola and Hakoköngäs, 2020). Adopting this particular idea of happiness, these readers are able to downplay the importance of well-known problems plaguing Finnish society, which are nevertheless acknowledged (‘of course, every country has their problems’).

### Social representations in force: Functions of anchoring and objectification

As we have sought to demonstrate, the anchorings and objectifications presented are not a mere description of how the WHR and the concept of happiness are made sense of by our actors, but rather serve as tools to provide justifications for the stances adopted towards Finland’s performance in the report.

Digital media adopt an ambivalent stance towards both the WHR and the concept of happiness. In other words, the same narrative is paradoxically aligned with both glorification and scandalization (Landahl, 2018) of the WHR. Glorification is enacted through the use of the sports news metaphor and by participating in the discourse around the secrets behind ‘Finnish happiness’. On the contrary, elements of scandalization are carefully introduced through systematic references to negative aspects of Finnish society and the construction of a concept of happiness as complex. We suggest that this ambivalent stance allows for the coexistence of a ‘range of interpretations’, leaving readers with the freedom to form their own understandings.

Being less preoccupied with creating compelling rhetoric or appealing to a wider audience, readers are more neatly divided, based on agreement or disagreement with the WHR.

When expressing support for the WHR results, our analysis shows that the report itself and the way it measures happiness are deemed of little relevance, and major emphasis is placed on Finland and its assets. The images circulating in this discourse (mythical places, winning the lottery and water) appeal to national identity and construct a more observable portrayal of Finnish society as functioning well.

In contrast, readers expressing criticism towards the WHR results are faced with the challenge of having to defend their portrayal of Finland in terms of dystopia against a ranking which proves the opposite. In line with previous studies (Rautalin, 2018), we suggest that in order to justify their dissatisfaction with the status quo, these readers need to employ more creative rhetoric, compared with those readers rejoicing at the WHR outcome. Our analysis shows that there are three different types of argumentations in disfavour of the WHR, which are based on specific objectifications: Finland being under an impending threat, bread queues and dictatorships.

Similarly to the modes of challenges to scientific claims introduced earlier (Barnes et al., 2018; Gierth and Bromme, 2020), the argumentations presented by our readers are addressed to the researchers themselves, their competence and their motives, on one hand, and to empirical aspects of the report, on the other. More specifically, following the model put forth by Gierth and Bromme (2020) (attacks on expertise, motivation, research methods and the thematic complexity of the topic), our analysis detected that similar types of justifications were adopted by our readers. First, the competence/expertise of the researchers are addressed in various ways, for example, through advancing doubts regarding their (un)awareness of Finnish culture and the way Finns are prone to social desirability when presented with a Likert-type-like scale. Second, the argument of motivation is made concrete through the use of dictatorship metaphors, which imply that the researchers behind the WHR benefitted from colluding with a malicious government by putting forward favourable ranking results. The argument questioning research methods is made concrete through the metaphor of the bread queue, which opens the stage for concerns regarding the construction of the sample and other methodological aspects. Finally, attacks towards the thematic complexity of the topic were evinced in the way some readers – and, to some extent, digital media – endeavoured to represent happiness as a ‘complex concept’ encompassing emotions and as being of a fleeting nature.

In addition to recognizing the four types of attacks on scientific claims set forth by Gierth and Bromme’s model, we suggest that the nature of the object of the report question – happiness – propelled our actors to devise a *fifth* way of attacking the WHR: eliciting fear of the status quo and imputing negative connotations to Finns and Finnishness. Unlike the other types of arguments presented, which in line with the previous literature either aim at attacking *empirical evidence* or *researchers*, this argument specifically aims at directly involving the *population* which the report is supposed to represent: Finns. In other words, this fifth type of argument aims at devaluing Finnish society by describing it in terms of a dystopia, and at devaluing Finns. This is intriguing, because this way of challenging scientific results seems to break from the typologies of challenges towards scientific claims identified so far in the literature.

To sum up, this study offers four main contributions to research on public understanding of science. First, the study further expands the typologies of challenges towards scientific claims identified so far in the literature (Barnes et al., 2018; Gierth and Bromme, 2020), suggesting that in addition to being directed at the scientific claim or the expert behind the claim, challenges can also be directed at research participants. Second, from a methodological perspective the study proposes a possible analytical strategy for exploring the construction of public understanding of science employing naturally occurring data retrieved from digital platforms.

Third, by focusing on the reception of a ranking measuring happiness, our study increases our knowledge of the public understanding of an abstract and fuzzy issue that belongs to the field of social sciences, a domain that according to Kronberger (2015) has received less scholarly attention compared with the public understanding of issues that are more easily quantified with standardized measures.

Finally, approaching public understanding of science from a Social Representations perspective, has enabled us to shed light on how common sense can challenge how (and why) research on happiness should be conducted. While the majority of studies adopting the SRT framework have mainly focused on how common sense ‘reformulates science’, considerably less attention has been given to the reverse relationship (Foster, 2003; Kronberger, 2015). The present study attempts to move towards this direction. More specifically, the multiplicity and richness of the public discourse around the WHR and happiness hint that part of the discontentment with the reports’ result might be due to the name the United Nations has chosen to label the entity measured in the ranking. We can hypothesize that, had the United Nations used the label ‘World Life Satisfaction Report’, the reception of the report would have been different among the ‘winners’ as well as internationally. Similarly to how names given to technologies affect the ways these are understood and made sense of among laypeople (Boersma et al., 2019), we suggest that better clarity in the way the happiness and proxies are operationalized by researchers is of uttermost importance when releasing studies that generate publicity and responses by the general public.

## 5. Conclusion

In the present study, we have shown how discussions on the WHR taking place among the ranking’s ‘winners’ can be treated as an opportunity to explore how the general public *spontaneously* challenges and problematizes the empirical concept of happiness and its measurement.

As the famous quote goes, ‘the only thing worse than a sore loser is an ungracious winner’, meaning that positive performances followed by scandalization are naturally bound to raise questions and demand clarifications.

Can we conclude that, at least for the large part, Finns are simply bad winners?

Our results indicate that in the public understanding of science, rankings are a specific type of scientific publication. The act of ranking different countries in regards to a certain measurable entity, anchored to the notion of winning sports contests in the media, inevitably stirs elements of national identity and an appraisal of one’s level of contentment with their nation’s *status quo*. When such appraisal is unfavourable, people justify their disagreement with the ranking by focusing their critiques on the research and/or on the researchers (Gierth and Bromme, 2020) but also on the research *participants*, who are constructed in terms of being either victims or villains belonging to a ‘dystopian’ society.

In addition, if we accept rankings as particular instances of scientific publications, the WHR is still a particular kind of ranking, as it measures a phenomenon on which the average citizen with no previous statistical knowledge can be considered expert. Our results show that agreement, disagreement, or ambivalence towards a ranking measuring happiness activate different dimensions of the everyday understanding of happiness, which are used to reinforce and justify the stance taken towards such ranking.

The present study is not without limitations, especially as regards the naturally occurring data on which we have based our analysis. First, when analysing readers’ comments on online platforms, it must be noted that readers do not simply respond to the news they are commenting on, but possibly also to other readers’ comments. Our research design, while proving fruitful for capturing highly shared processes through which representations of the WHR and happiness are created, did not equip us with the fine-grained analytical tools needed to capture the interlacing aspects characterizing interactions that unfold among readers. Second, it has been previously hypothesized (Peters and Dunwoody, 2016) that the motivation to comment on an online article tends to be higher if readers disagree with the claim of the news story, compared with when they agree. Thus, while we have proposed that the justifications of readers disagreeing with WHR appear particularly creative and elaborate, it is reasonable to suspect that readers maintaining a negative stance towards the publication might be overrepresented among the online comments. We urge other scholars to continue to follow the reception of the WHR and similar reports generating considerable publicity, also taking into account countries occupying different positions in the ranking, in order to investigate possible similarities with and differences from the ‘winners’.

## Supplemental Material

sj-docx-1-pus-10.1177_09636625221132380 – Supplemental material for Are we bad winners? Public understandings of the United Nations’ World Happiness Report among Finnish digital media and their readers Click here for additional data file. Supplemental material, sj-docx-1-pus-10.1177_09636625221132380 for Are we bad winners? Public understandings of the United Nations’ World Happiness Report among Finnish digital media and their readers by Jennifer De Paola and Anna-Maija Pirttilä-Backman in Public Understanding of Science

## Notes

1. While these examples (Rautalin, 2018; Steiner-Khamsi, 2003) relate specifically to international rankings measuring performance in the field of education, we propose that presenting an overview of the rankings in this field is relevant due to the attention received by the Programme for International Students Assessment (PISA) survey in Finland.

2. For more detail on the societal position of these news forums, see Lounasmeri and Ylä-Anttila (2015) and Venäläinen (2016).

3. Translations of the presented excerpts from Finnish to English were carried out by the first author.

4. Analysis dealing with the online readers’ comments will appear lengthier than analysis of digital media. Due to readers presenting themselves as neatly divided into two groups based on their agreement or disagreement with the World Happiness Report (WHR), more space was needed to present an exhaustive account of both groups.

5. In popular Finnish culture, this refers to the anthropomorphic representation of Finland in the form of a young maiden, whose silhouette is seen in the shape of Finland on a map.

6. German Democratic Republic.
