# Supplementary material for: Are we bad winners? Public understandings of the United Nations’ World Happiness Report among Finnish digital media and their readers
Source: Public Underst Sci. 2022 Dec 5;32(1):20–39. doi: 10.1177/09636625221132380 (PMC9814021; doi:10.1177/09636625221132380)
Supplement: sj-docx-1-pus-10.1177_09636625221132380 – Supplemental material for Are we bad winners? Public understandings of the United Nations’ World Happiness Report among Finnish digital media and their readers [file sj-docx-1-pus-10.1177_09636625221132380.docx]

**Supplemental material for the article “Are we bad winners? Public understandings of the United Nations’ World Happiness Report among Finnish digital media and their readers.”**

Jennifer De Paola ^a^

Anna-Maija Pirttilä-Backman ^a^

^a^ University of Helsinki, Finland

Corresponding author: Jennifer De Paola,

Unioninkatu 33, 00170 University of Helsinki, Finland.

Phone: 00358 40 5012016, e-mail: jennifer.depaola@helsinki.fi

**SUPPLEMENTAL MATERIAL**

In the text below, we illustrate in more detail how we combined basic tenets of Grounded Theory with conceptual tools derived from Social Representation Theory.

***Data collection and coding process***

The process of data collection followed basic Grounded Theory guidelines (e.g. Corbin and Strauss, 1990) which entails starting the analytical process while the data collection is still ongoing. Following these guidelines, the analysis started from the readers’ comments regarding the WHR from two digital media sources (Helsingin Sanomat and Yle) which were read through carefully until we acquired a general understanding of how to proceed with the data.

Careful reading was followed by labelling interesting and recurring words, phrases and concepts (open coding), which made us notice that the comments were situated in open dialogue with the corresponding online news articles. For this reason, we further expanded the data collection to the news articles corresponding to the comments and continued the coding. We either produced codes by coming up with words or sentences that we thought best described the segment of text or by using words/sentences already present in the data (in vivo coding technique).

At this point, we observed that the journalists often mentioned how other Finnish tabloids were presenting the WHR. Following this hunch, we again expanded the data collection, this time including news articles and related comments from two tabloid-type digital media sources (Ilta-Sanomat and Iltalehti) and continued the coding process.

The phase of open coding generated a large amount of codes, including codes that were not relevant to the present study, i.e. investigating the public’s understanding of the WHR and happiness. Thus, we proceeded to set aside unrelated codes while simultaneously comparing the emerging list of codes with the data we were working with. We then grouped codes into tentative categories, creating sub-categories as needed. For example, the main category ‘negative scenario’ shown in the example below, grouped together all the codes that shared the similarity of portraying a picture of Finland characterized by negative connotations.

At this point, we started exploring the different ways in which categories relate to each other (axial coding). This phase was complemented with the analytical tools afforded by SRT, which entailed searching for objectifications and anchoring within emerging categories as shown below.

| **EXAMPLE**  Suomineito käyskentelee synkässä metsässäkin suorastaan lapsellisen oloisesti huomaamatta puun takan kyyristelevää vihaista pentujaan suojelevaa karhua, joka iskee heti tilaisuuden tullen. (HS, 2018)    ENG: The Finnish Maiden is walking in a childish way in the gloomy forest, without noticing the angry bear crouching behind the tree, ready to attack the maiden as soon as the opportunity arises to protect her own cubs.    Coding process: bear crouching behind the tree ready to attack > impending threat (code)> negative scenario (main category)      Searching for anchoring and objectifications :  ⮚ dangerous beast (objectification)  ⮚ Finland as ‘almost dystopia’ (anchoring) |
| --- |

# **Searching for anchoring and objectifications**

When searching for objectifications, we looked at all the images, icons, symbols, and metaphors used to lend concrete forms to the otherwise abstract or complex concepts. Objectifications were identified directly from text (or images). They were often highly shared among readers and journalists.

We considered anchoring in terms of a process involving naming and classifying novel or complex phenomena within the backdrops of existing understandings and classifications. Anchorings could be extracted directly from text or presented as the result of our interpretation. In the example shown above, the code ‘impending threat’, together with other codes grouped in the main category ‘negative scenario’, were interpreted as being anchored to the notion of ‘dystopia’, a concept that in popular culture represents a society characterized by misery, decay, squalor, and oppression.

It is to be noted that although presented as separate phases, ‘coding process’ and ‘searching for anchoring and objectifications’ did not constitute a linear process. On the contrary, refining categories often required going back and forth between codes and the emerging main categories, testing codes produced initially and adjusting correspondent categories.
